# First report of molecular identification of *Phytophthora infestans* causing potato late blight in Yemen

**DOI:** 10.1038/s41598-023-43510-2

**Published:** 2023-09-29

**Authors:** Amira A. Al Harethi, Qais Y. M. Abdullah, Hala J. Al Jobory, Samar A. Al Aquil, Ramadan A. Arafa

**Affiliations:** 1 Present Address: Department of Biological Science, Faculty of Science, Sana’a University, Sana’a, Yemen; 2 Department of Physics, Faculty of Science, Sana’a University, Sana’a, Yemen; 3 Plant Pathology Research Institute, Agricultural Research Center, Giza, 12619 Egypt

**Keywords:** Microbiology, Plant sciences

## Abstract

Late blight, caused by *Phytophthora infestans*, is one of the most destructive potato diseases in the world. In Yemen, identification of *P. infestans* still depends on a visual survey and external examination of late blight symptoms. The objective of this study was to isolate and identify *P. infestans* by using advanced methods. We collected 71 disease samples and isolated the pathogen using the tuber slice method. To identify an isolated pathogen, we performed morphological characterization and gene sequence analysis of the coding genes for internal transcribed spacers. We used Koch’s hypotheses to confirm the previous results. In our study. The morphological characters of the mycelium pattern of *P. infestans* isolates in Yemen were profusely branching, fluffy, and white. The sporangia showed remarkable limoniform papillate sporangial shape. with average length and width of 30.6 and 28.6 µm, respectively. The sequences analysis showed high homology with a degree of identity ranging from 98 to 100% to the database sequences on GenBank. Pathogenicity tests showed that* the P. infestans* was the causal agent. To our knowledge, this is the first study of the isolation and characterization of *P. infestans* in Yemen.

## Introduction

Potato (*Solanum tuberosum* L.) ranks third in terms of human consumption, trailing rice and wheat^1^. It is also the world's most important non-grain food product^2^. In terms of nutritional value, potato contains essential nutrients such as potassium, vitamin C, vitamin B6, dietary fiber, and magnesium. Globally, potato is a significant staple food and has a better overall nutrient-to-price ratio than many other vegetables^3^. According to the latest FAOSTAT data, which was updated^.^ In 2021, Yemen was estimated to produce 228,352 metric tons of potatoes^4^, which is 0.02% less than the 233,201 tons produced in 2020^5^. In 2022, Yemeni Ministry of Agriculture and Irrigation bans importing potato seeds for achieving local sufficiency^6^ Yemen has previously imported potato seeds primarily from the Netherlands, France, and Oman^7^.

Late blight of potato, caused by the phytopathogenic oomycete *Phytophthora infestans* (Mont.) de Bary, is one of the most destructive potato diseases in the world^8^. The annual worldwide potato crop losses due to late blight was estimated to exceed $6.7 billion^9^.

*P. infestans* has two mating types, A1 and A2, that are needed for sexual reproduction. The A1 mating type was dominant in the global population of *P. infestans* until the 1980s, when the A2 mating type was introduced from Mexico and spread rapidly to many regions. The coexistence of both mating types increased the potential for sexual reproduction and oospore formation in *P. infestans*, posing a serious threat to potato and tomato production worldwide^10^.

Identifying fungi is an essential step in understanding their role in disease development and management. Different approaches have been developed to identify fungal species, ranging from morphological characteristics, cultural features, biochemical tests, and serological techniques to advanced molecular biology tools such as polymerase chain reaction (PCR), DNA sequencing, and bioinformatics analyses^11^. It is becoming increasingly evident that accurate and useful data related to fungal identification and authentication can only be obtained through a multistep approach. Therefore, it is necessary to incorporate both traditional methods and modern molecular biology techniques^12^. Recently, a new approach known as the polyphasic approach has been developed for reliable identification and characterization of fungi. This approach involves the utilization of different techniques based on the organization of scientific knowledge^13^.

In Yemen, the characterization of the *P. infestans* population is still largely unknown, and to our knowledge, most of the previous studies on *P. infestans* relied on a visual scan survey and external examination of late blight symptoms, including Kamal and Agbari^14^. The goal of this study was to isolate and identify *P. infestans* using morphological characterization and molecular identification. We used Koch's hypothesis to confirm these results. To the best of our knowledge, this is the first study to isolate and characterize *P. infestans* in Yemen.

## Results

### Observation of potato late bight symptoms in Yemen

**Figure 1:**
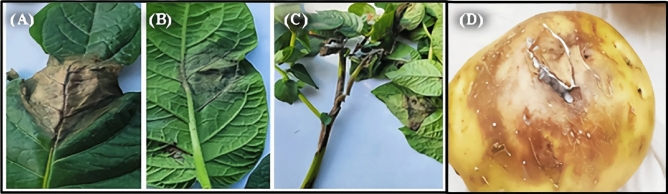
Late blight symptoms on different parts of potato plant. (**A**) Potato leaf (uper side) (**B**) The abaxial (lower) side of the leaf, (**C**) Potato stem and (**D**) Potato tuber.

Favorable environmental conditions allow the late blight pathogen to establish quickly within fields and destroy potato plants. Visual observation of late blight symptoms, which are often water-soaked, brownish-black, and irregularly shaped lesions on the edges of the leaf, and white growth (sporulation) evident on the abaxial surface of the leaf lesions, is the first step toward the detection of *P. infestans* at early stages of infection (Fig. 1A,B). Stems can also exhibit dark brown to black lesions with sporulation (Fig. 1C). Symptomatic tubers typically have sunken and firm brown lesions that may extend several centimeters into the tuber. The variability in lesion appearance is often the result of differences in moisture. Infected tubers develop a firm brown decay that starts on the outside and may later extend to include the outer 0.125 inch (3–12 mm) of tissues (Fig. 1D).

Potato late blight was surveyed on different fields of the Yerim/Ibb governorate during the growing season of 2021. A total of 54 leaves and 17 stems, which were collected from 5 fields, were used in this study (Table 1).

**Table 1:** Yemen's geographic regions where symptoms of late blight were collected.

No	Isolate name	Governorate	District	Host/part	Number of collated samples	Sampling year
1	YE_1	Ibb	Yerim	Potato stem	17	2021
2	YE_1.2		Yerim	Potato leaves	54	2021
Total		1	1	1/2	71	1 year

### Morphological characterization

**Figure 2:**
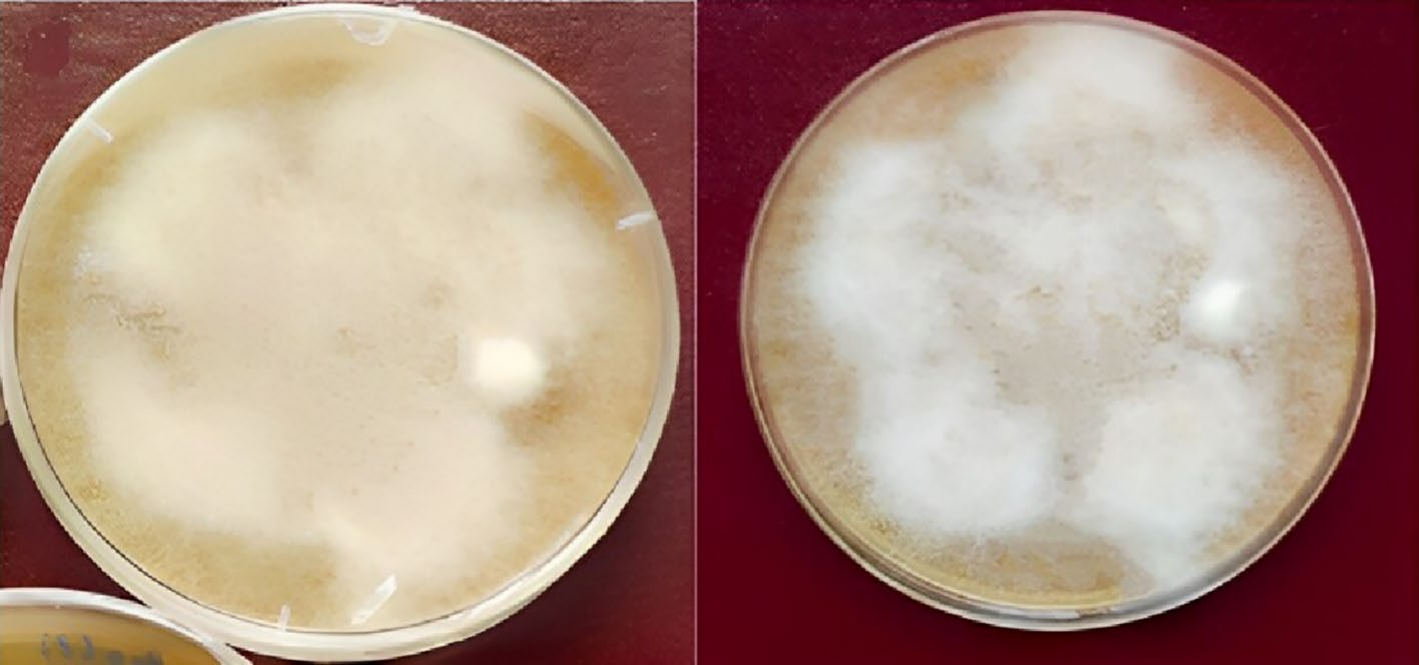
Mycelial growth of *P. infestans* grown on V8 agar media.

The morphological characteristics were evaluated by using seven-day-old and 14-to-21-day-old cultures of *P. infestans.* The radial growth obtained on V8 agar was 76 mm (Fig. 2). The macroscopic and microscopic characters of the mycelium pattern of *P. infestans* isolates in Yemen were profusely branching, fluffy, and white (Fig. 2A).

**Figure 3:**
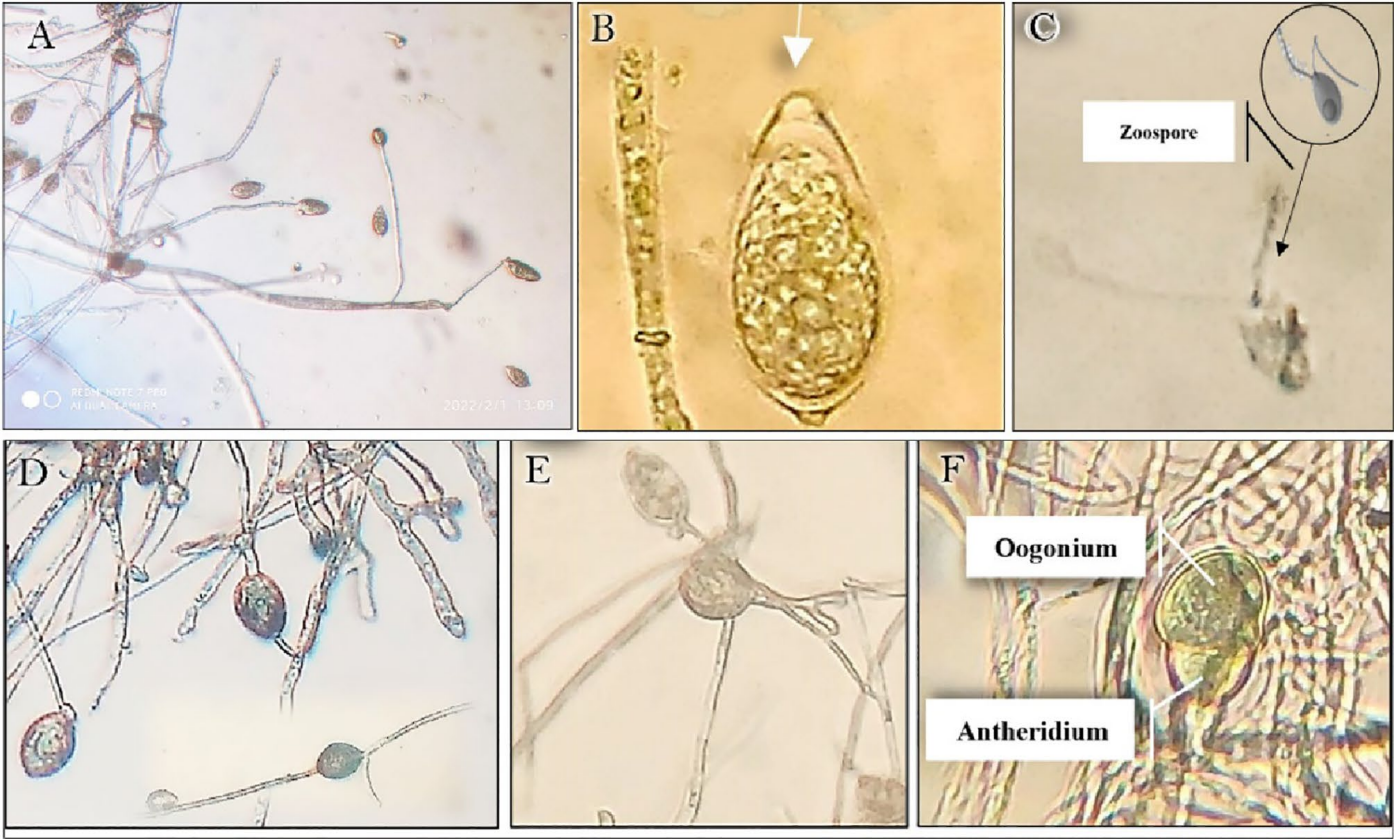
Morphological characteristics of *P. infestans* grown on V8 agar media (**A**) Terminal sporangia (**B**) limoniform sporangia , with a papilla at the distal end in. (**C**) Biflagellated zoospores. (**D**) Intercalary Chlamydospores (**E**) A germination cyst (**F**) Amphyginous antheridium with Spherical to obpyriform oogonium.

These mycelia were aseptate. Morphological characters of sporangia revealed terminal sporangia (Fig. 3A) with a remarkable limoniform papillate sporangial shape (Fig. 3B). The liberated zoospore is a biflagellate and uninucleate structure (Fig. 3C). In addition, terminal and intercalary chlamydospores (“asexual thick-walled spores”) appeared in the current study (Fig. 3D), where the strong walls of these peculiar spherical structures distinguished them from hyphal swellings. Furthermore, when the conditions are favorable, chlamydospores germinate into sporangiophores containing sporangia (Fig. 3E). The antheridium was amphigynous with Spherical to obpyriform oogonium (Fig. 3F). Sexual reproduction and self-fertile phenomena have been seen in the *P. infestans* population in Yemen (Fig. 3F).

**Figure 4:**
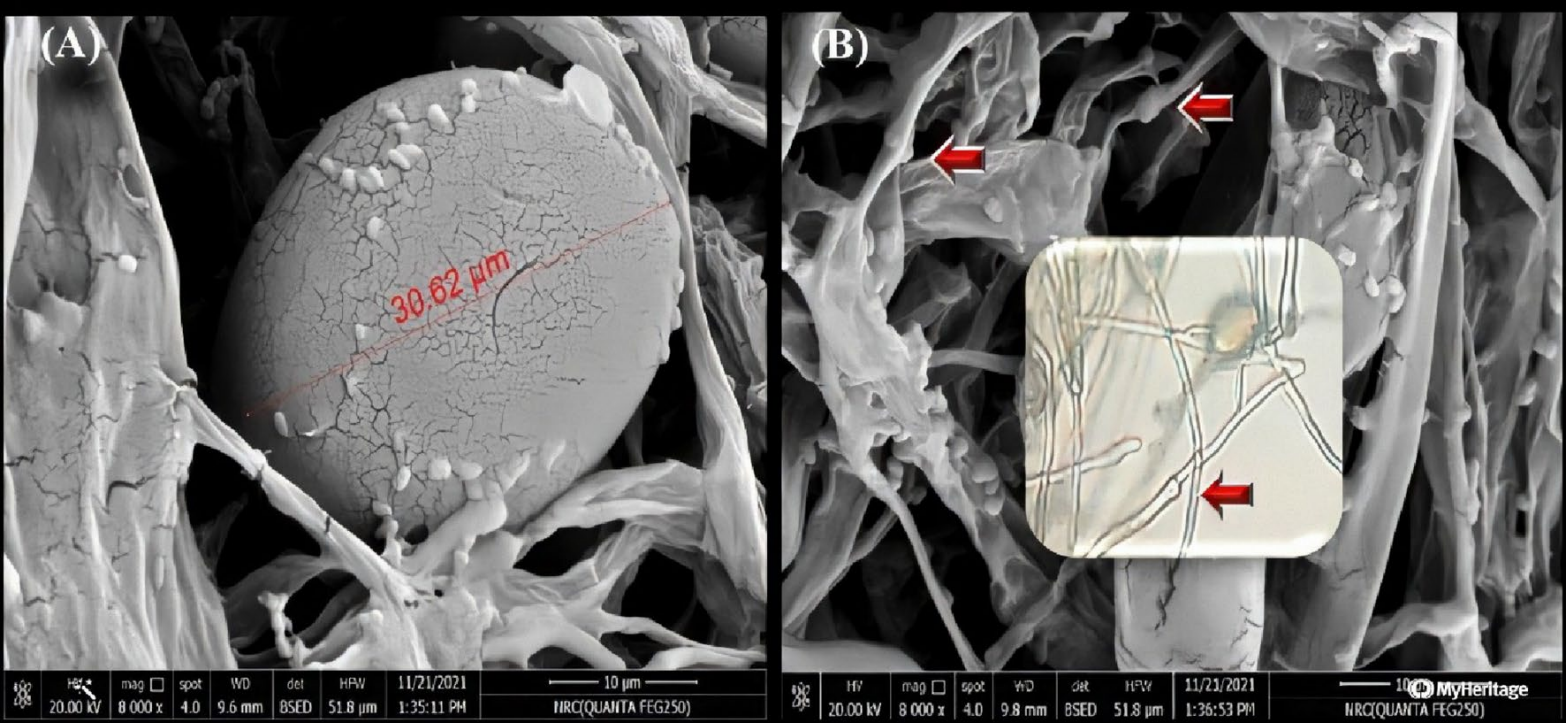
Features of *P. infestans* using scanning electron micrographs (**A**) A lemon-shaped sporangia 30.6µm in diameter that produces zoospores. (**B**) Arrows indicate *Phytophthora* apophyses in sporangiophores as seen in SEM and light microscopy. The following parameters were used to establish electron micrographs: 20.0 kV, 8000x, and 10µm for both images.

After 2–3 weeks, *P. infestans* isolates demonstrated different morphological features under compound and scanning electron microscopes. The average length and width of sporangia were 30.6 and 28.6 µm, respectively (Fig. 4A). Furthermore, sporangiophores differ from mycelia in that they are upright, branched, complex, and sympodial, with a slight distinctive bulge at the base of each branch (Fig. 4B).

### Molecular identification and phylogeny

**Figure 5:**
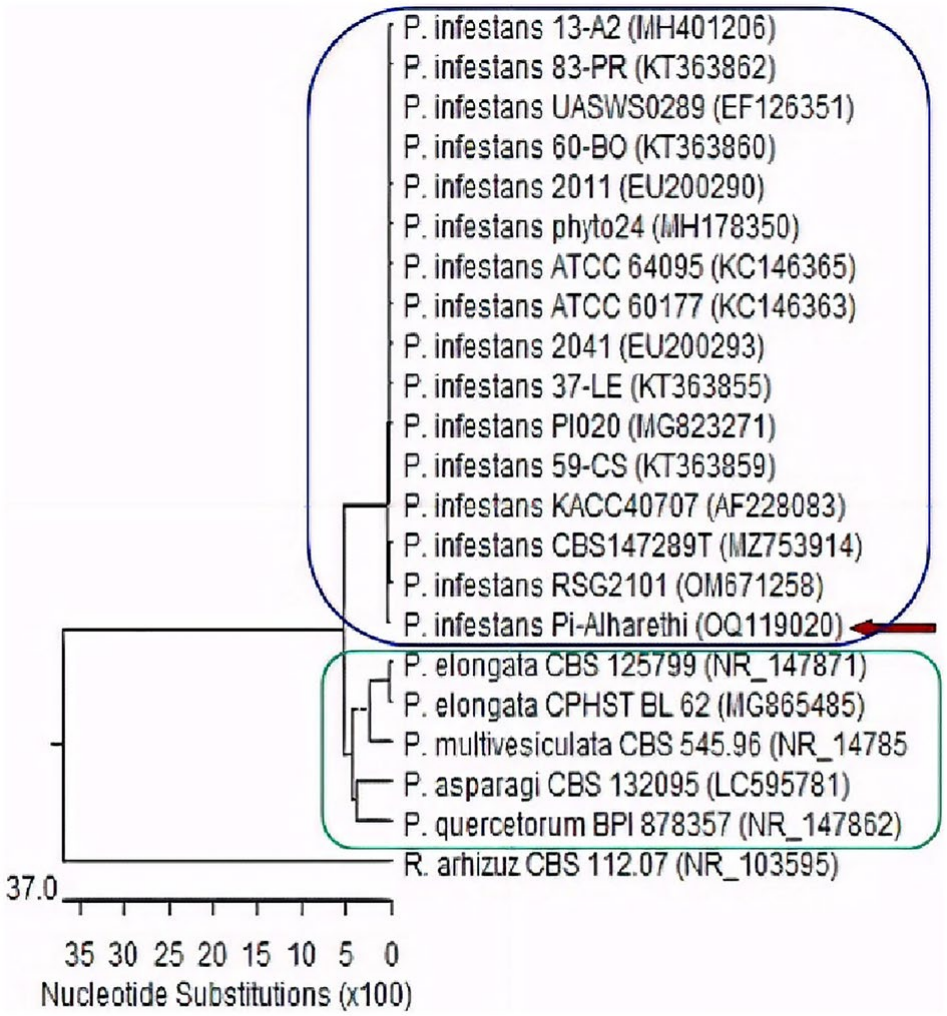
Maximum likelihood Phylogenetic tree of *P. infestans* Pi_Alharethi_YEM2021 isolate based on ITS sequences, with a bootstrap value of 1000 repetitions compared to other sequences retrieved from GenBank. Rhizopus arhizuz is the outgroup strain. P. = *Phytophthora*; R. = *Rhizopus.*

BLAST analysis of the sequence of *Phytophthora infestans* Pi_Alharethi_YEM2021 showed high homology with a degree of identity ranging from 98 to 100% to the database sequences on GenBank A phylogenetic tree was designed to represent the evolutionary connection between the identified strain and other species (Fig. 5). Our strain was found to be in a cluster with other submitted *Phytophthora* isolates from GenBank. According to phylogenetic analysis, the sequences of *Phytophthora* isolate were found in different subclusters but constituted the same cluster as *P.* elongata, *P. multivesiculata, P. asparagi,* and *P. quercetorum*. In the dendrogram, *Rhizopus arhizuz* established a distinct cluster (outgroup). We found that the ITS region of the isolate obtained from Yerim revealed sequence homology with isolates previously described from other regions, including isolates from United Kingdom^15^, France^16^ and Mexico^17^.

### Pathogenicity of an isolated pathogen

**Figure 6:**
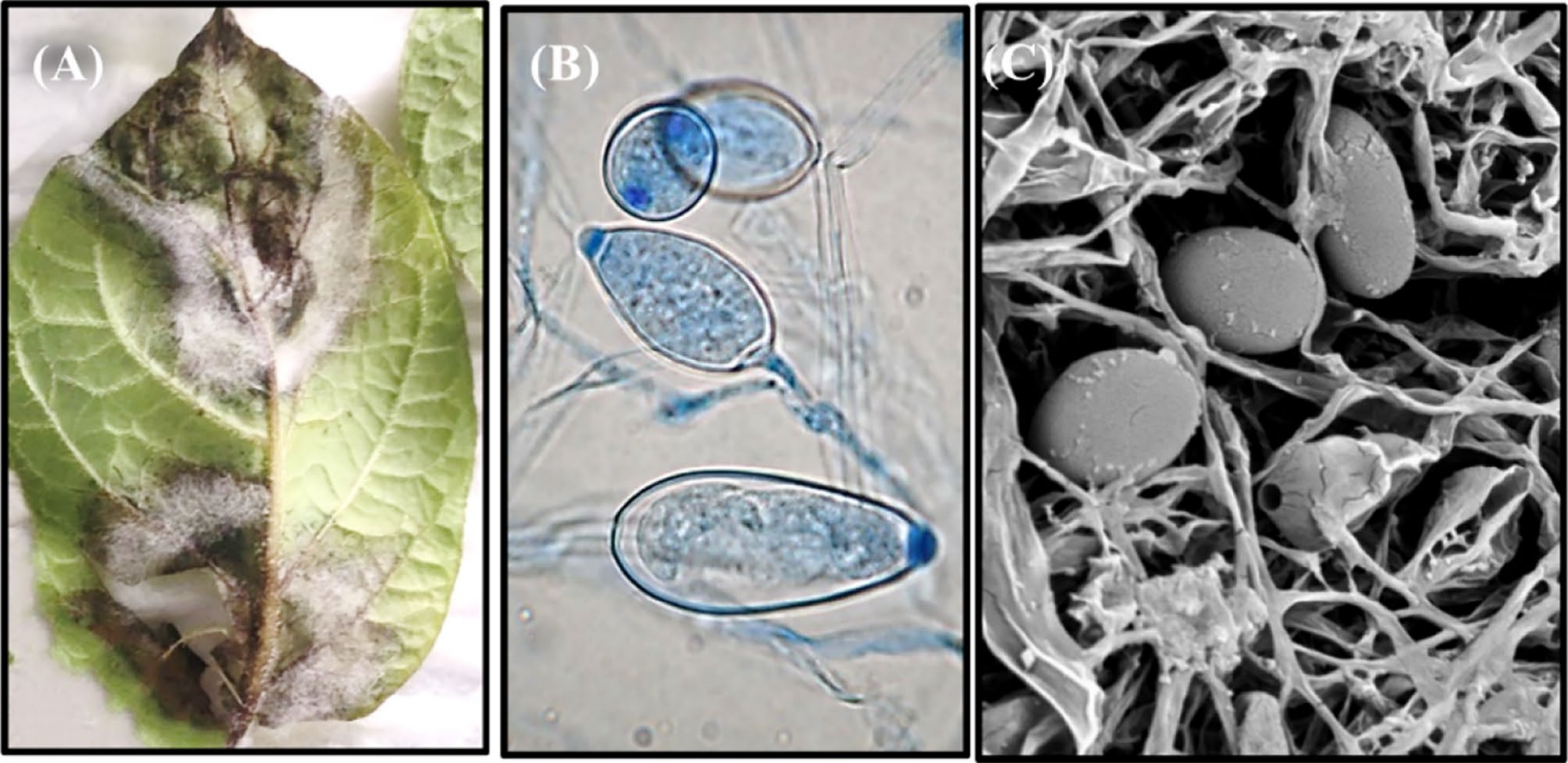
(**A**) Pathogenicity test of *P. infestans* showing late blight symptoms on potato leaflet Micrography of *P. infestans* showing sporangia under (**B**) Light microscope and (**C**) Scanning electron microscope.

A pathogenicity test of all inoculated isolates was performed, with monitoring the symptom. After 10 days of inoculation, visual observation of late blight symptoms revealed water-soaked, brownish-black, and irregularly shaped lesions on the edges of the leaf and white growth (sporulation) evident on the abaxial surface of the leaf lesions (Fig. 6A). The isolated pathogen was examined microscopically, and the findings were compared with those detected under natural infection conditions and were found to be identical (Fig. 6B,C). Based on these findings, *P. infestans* is considered the causal agent of potato late blight in Yemen.

## Discussion

For effective crop and pest/pathogen control techniques, precise detection and identification of causative pathogens of agricultural plants is essential. In this investigation, the *Phytophthora infestans* strain Pi Alharethi YEM2021-caused late blight disease was first identified in Yemen. As a result, the exact identification of the agent causing late blight was highlighted in this study. *P. infestans* was isolated and identified based on morpho-molecular characterization, in which macroscopic examination and microscopic analysis for characteristic fungal structures, in addition to gene sequence analysis of the coding genes for internal transcribed spacers (ITS), were performed using the appropriate primers. As a result, *P. infestans* was identified as the main causative agent of late blight of potato and tomato using rDNA spacer sequence and BLAST analysis. The obtained results are in agreement with Erwin and Ribeiro^18^. who described in detail the pathogen *P. infestans* by illustrating the sporangiophores and sporangia in which mycelium appeared coenocytic, multinucleated, and aseptated. The sporangium was opaque, lemoniform, and had a papilla at the distal end, as mentioned by Gevens and Seidl^19^.

Our results demonstrated the ability of *P. infestans* to produce chlamydospores, as confirmed by a few similar studies^20^. The existence of chlamydospores in the fields has been shown to be a source of soil-borne *P. infestans* inoculum, in which they are created throughout the winter and serve as a cause of infection for the life cycle of the next season^20^.

In our study, we couldn't get reference isolates to determine the presence of mating types in *P. infestans*. However, in the current investigation, the production of oospores in a single-pure culture of Yemeni isolates confirmed the presence of homothallic (self-fertility) within the *P. infestans* population. In homothallic species, oospores form with a single mating type. Furthermore, Zhu et al.^21^ have revealed that these self-fertile isolates pose a new threat to potato and tomato crops due to their enhanced genotypic diversity of the pathogen population and exhibit higher aggressiveness and greater fungicide resistance than A1 isolates. Moreover, homothallic isolates of *P. infestans* have been identified in Mexico^22^, China^23^, and Egypt^24^. Moreover, a study conducted in Algeria demonstrated that both A1 and A2 mating-type isolates *of P. infestans* were detected among Algerian isolates in percentages of 70% for the A2 mating type and 30% for the A1 mating type^25^. In contrast, research conducted in Egypt revealed that the A1 mating type predominated among Egyptian isolates^26^.

Regarding pathogen characterization, the pathogenic capability of *P. infestans* strain Pi Alharethi YEM2021 was evaluated by Koch's hypothesis using detached-leaf assay. The test pathogen was extremely aggressive and showed recognizable symptoms such as water-soaked, irregularly shaped, brownish-black lesions on the margins of the leaf. White growth (sporulation) was also seen on the leaf's abaxial surface. These findings offer genetic and geographic data for planning a Yemeni late blight disease management strategy.

Internal transcribed spacer (ITS) amplification using genus and/or species specific ITS primers and PCR were used in the current investigations to successfully identify *P.* *infestans*. As a result, *P. infestans* was identified as the main causative agent of late blight in potato. The evolutionary link between the identified strain and other species was represented by a phylogenetic tree which showed dominance in *P. elongata, P. multivesiculata, P. asparagi, and P. quercetorum*. The evolutionary link between the identified strain and other species was represented by a phylogenetic tree.

## Materials and methods

### Sample collection

**Figure 7:**
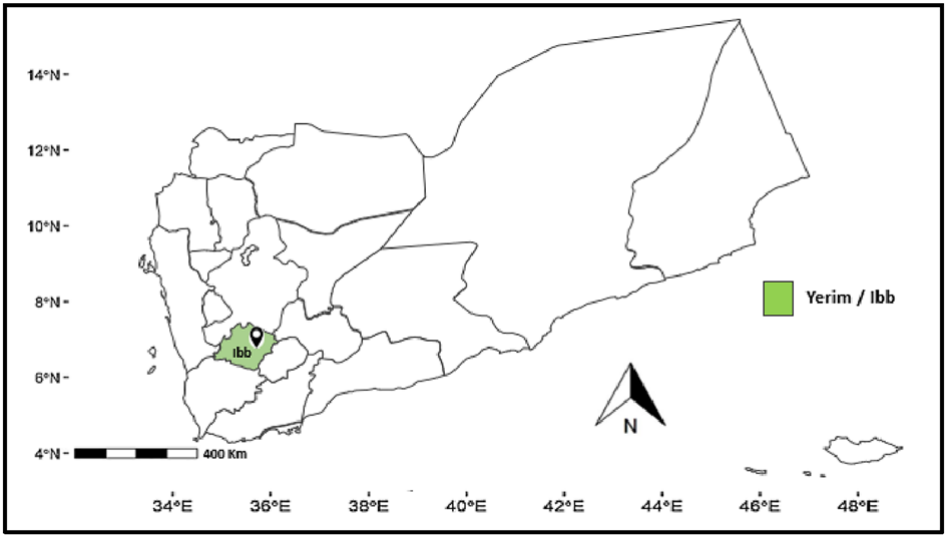
Late blight sample collection Ibb area (Yerim), Yemen.

During the growing season of 2021, visibly infected leaves and stems with distinctive symptoms of late blight were picked up from potato plants. The blighted samples were collected from the areas of the Seed Potato Production Center (SPPC) in Ibb Governorate in Yemen (Fig. 7). Our experimental research on plants; including the collection of plant material, complies with relevant institutional, national, and international guidelines and legislation. Leaves and stems with a single, fresh, nicely sporulating lesion were selected from four different plants/fields (two lesions from each plant). The collected fresh leaves and stems were transferred from the open field to the laboratory under cold conditions of − 4 °C^27^ in transparent polyethylene bags. A total of 54 leaves and 17 stems, which were collected from 10 fields, were used. A list of the 71 isolates used in this study, including isolate name, location of collection, host, and sampling year, is presented in Table 1.

### Isolation and purification of *P. infestans*

This trial was conducted at the laboratory of microbiology, College of Science, University of Sana'a, Yemen. Isolation of *P. infestans* was conducted using the tuber-slice method as described by Tumwine et al*.*^28^. The collected leaf samples were carefully washed under tap water in order to remove the dust, insect eggs, and soil remains, and then dried on Whatman filter paper at room temperature. Moreover, healthy potato tubers were sterilized by rinsing in 1% sodium hypochlorite (NaClO) for 2 min and were transferred to Whatman filter paper at room temperature for drying. Subsequently, blighted potato leaves were cut into small pieces and were placed into healthy, sterilized potato tubers in a Petri dish, which were incubated at 18°C in darkness for seven days for sporulation enhancement. A thick white growth of sporangiophore with plenty of zoosporangia covered the tuber slice surface within 5–6 days.

The white mycelium of *P. infestans* was picked up using a glass needle, and it was transferred to 9 cm Petri plates using V-8 agar medium (100 ml of V8 juice, 0.05 g ß-sitosterol, 1g CaCO3, and 15 g agar; the final volume was adjusted to 1 L with distilled water and autoclaved at 15 psi for 20 min) amended with 100 mg/L ampicillin, 20 mg/L rifampicin, and 50 mg/L nystatin^29^. The purified cultures of *P. infestans* were kept at − 4 °C^30^.

### Morphological characterization

To identify *P. infestans* isolates, the Laboratory Protocols of *Phytophthora* species from the American Phytopathological Society were utilized, according to Ivors^31^. This approach was utilized to develop important morphological features, including colony morphology, sporangium and oospore development. Moreover, scanning electron microscopy was used in accordance with Bozzola^32^. This method allows for the study of microbes on the substrate's surface, hyphae morphology, and spore production.

We evaluated the morphological characteristics of the isolated *P*. *infestans* after 7 days of incubation at 18 °C in the light. After 2–3 weeks of incubation of single cultures at 20 °C in the dark, we investigated the presence of chlamydospores, spore production, and sporangial morphology of the isolated *P. infestans*.

Samples prepared by tacking a disc with approximately 0.5 cm of a solid medium containing mycelia of *P. infestans* were mounted on copper stubs (approximately 2.5 × 2.5 cm) in a double carbon tape pasted on an aluminum foil film. In a sputter, the specimen was coated with gold for 5 min, then, all morphological characters were recorded. *P. infestans* micrography was taken under scanning electron microscopy and magnification of 8000 × (SEM QUANTA-FEG 250 with field emission gun, FEI Company Netherlands) at the National Research Center, Egypt.

### Extraction of DNA, PCR Amplifcation and Sequencing

*P. infestans* isolate was cultured on V8 medium and incubated at 18°C for two weeks^33^. At Assiut University's Molecular Biology Research Unit, fungal DNA was extracted using a Patho-gene-spin DNA/RNA extraction kit (Intron Biotechnology Company, Korea). Sequencing and polymerase chain reaction (PCR) were carried out at the SolGent Company in Daejeon, South Korea. In order to amplify the ITS region of the rRNA gene, the universal primers ITS1 (forward) and ITS4 (reverse) were used. Primers have the following composition: ITS1 (5'- TCCGTAGGTGAA CCTGCGG -3′), and ITS4 (5′- TCCTCCGCTTATTGATATGC -3′). With the addition of ddNTPs to the reaction mixture, the purified PCR product was sequenced using the same primers^34^.

### Phylogenetic analysis

The obtained sequences were analyzed using the Basic Local Alignment Search Tool (BLAST) from the National Center for Biotechnology Information (NCBI) website. To determine phylogenetic relationships, the obtained sequences were aligned with closely related sequences of fungal strains accessed from GenBank using Mega 11^35^ where the MUSCLE algorithm was used, and a phylogenetic tree was constructed using the maximum-likelihood (ML) approach based on the Hasegawa-Kishino-Yano model 1985. The best model was selected based on the lowest Bayesian Information Criterion (BIC) scores using MEGA11. The initial tree(s) for the heuristic search were obtained automatically by applying the Neighbor-Join and BioNJ algorithms to a matrix of pairwise distances estimated using the Maximum Composite Likelihood (MCL) approach. A resampling procedure (bootstrap) with 1000 trials was used to validate the confidence of the clades. The analysis involved 22 nucleotide sequences. There were 956 positions in the final dataset.

### Pathogenicity test

Koch's postulates were applied to confirm that the isolated microorganism was the actual cause of late blight by using a modified detached-leaf assay as described by Karki^36^. Three plugs of *P. infestans* were cut and re-inoculated in a new (V-8) media plate in the dark at 18 °C. The plates were kept facing downward to avoid any moisture development on the plugs. The sporangia from a 10 -14 day old were taken off the plate by flooding the plate with 5 ml ice cold sterilized water (to expedite the release of zoospores) and mixing properly with a spreader. Then, the plate was kept at 4 °C for 2 to 4 h to release zoospores. Zoospores were collected by filtering through two layers of sterile cheesecloth and diluted in 20 ml of ice-cold, sterilized water. The motile zoospores were counted by using a hemocytometer and adjusted to about 50,000 (zoospores and water) with 50,00 zoospores from a 10 /ml under a microscope^36^.

**Figure 8:**
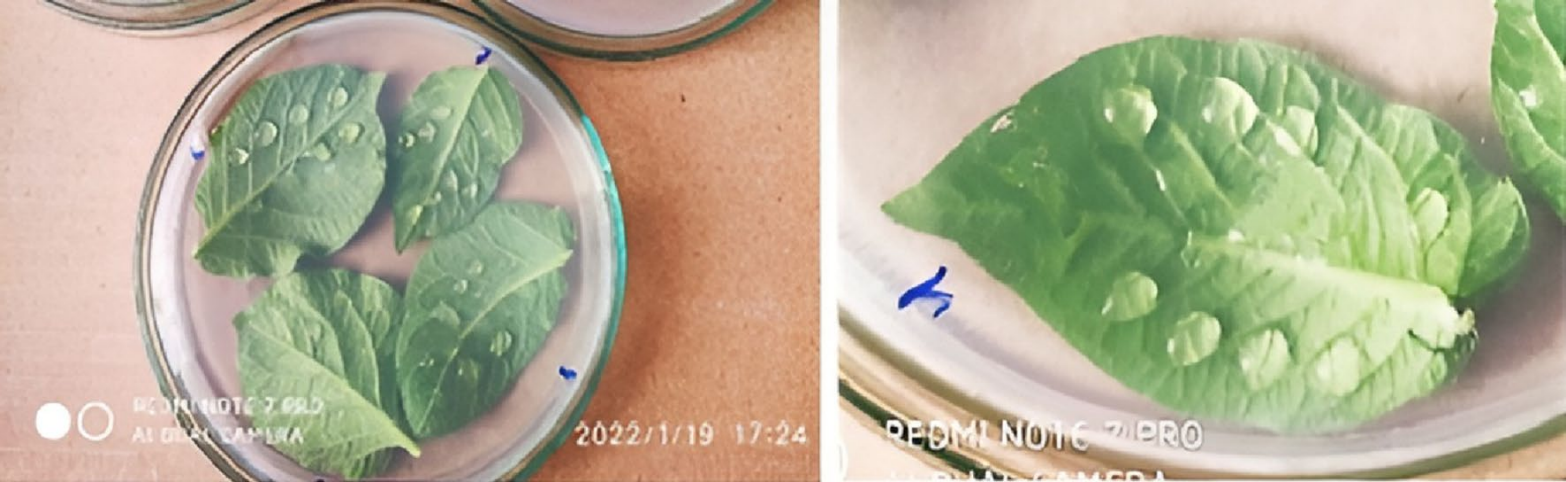
Detached-leaf assay for pathogenicity test of *P. infestans.*

Healthy, full-grown compound leaves from 6 to 10-week-old plants were collected. The abaxial side of each leaflet was inoculated with 4–6 droplets of inoculum (50,000 zoospores per ml) with an Eppendorf repeater and placed in a Petri dish filled with sterilized water agar media. Thereafter, the Petri dishes were incubated in a room with natural light at a temperature of 21 °C and monitored daily. After 5 days, the leaves were assessed visually for the appearance of symptoms (Fig. 8). Samples from the incubated pathogen were isolated for morphological characterization under scanning electron microscope.

## Conclusion

71 late blight isolates were gathered and isolated from potato leaves, and stems for the current investigation. The ability of isolates to cause illness and the occurrence of the specific side effects, such as yellowing, wilting, or plant death, were used to confirm the isolate's pathogenicity. To determine the related pathogen, the morphological properties of the causative pathogen were examined on both the host and an artificial culture medium. The ITS1-ITS4 region was studied using PCR sequencing, and the results revealed that the isolated pathogen was *Phytophthora infestans* strain Pi Alharethi YEM2021, which was successfully uploaded to GenBank under the accession number OQ119020 For the first time, *P. infestans* strain Pi Alharethi YEM2021 I incident has been recorded in Yemen.

This was a short-term study that focused on the identification and characterization of *P. infestans* in Yemen for the first time; consequently, long-term studies are needed to investigate the diversity of *P. infestans* populations in Yemen.

## Data Availability

The datasets generated and analyzed during the current study are available in the National Center of Biotechnology Information's (NCBI), The nucleotide sequences obtained with ITS has been uploaded successfully under the accession number GenBank OQ119020 website's, https://www.ncbi.nlm.nih.gov/nuccore/2417000057.
